# KANSAS’S PEAK 2.0: AN ACADEMIC-STATE PARTNERSHIP IMPROVING THE LIVES OF NURSING HOME RESIDENTS

**DOI:** 10.1093/geroni/igz038.2049

**Published:** 2019-11-08

**Authors:** Gayle Doll, Laci Cornelison, Robyn Stone

**Affiliations:** 1 Kansas State University, Manhattan, Kansas, United States; 2 LeadingAge, Washington, District of Columbia, United States

## Abstract

Most academic institutions welcome partnerships with industry and state government. These collaborations can lead to interventions to create social and environmental changes on a broad scale. Along with the opportunities, some challenges are inherent with these working relationships. The Kansas State University Center on Aging and the Kansas Department for Aging and Disability Services has been working together for more than 15 years on the Promoting Excellent Alternatives for Kansas nursing homes (PEAK) program. This collaboration has led to beneficial changes for nursing home residents and provided fertile ground for researchers wanting to examine these environments. This symposium will offer researcher insights as well as to elucidate process and procedures related to developing and maintaining collaborations with a state agency.

